# Dietary *Forsythia suspensa* extracts supplementation improves antioxidant status, anti-inflammatory functions, meat fatty acid deposition, and intestinal microbial community in finishing pigs

**DOI:** 10.3389/fvets.2022.960242

**Published:** 2022-10-14

**Authors:** Sujie Liu, Qianqian Wang, Jiayu Ma, Jian Wang, Hongliang Wang, Li Liu, Shenfei Long, Xiangshu Piao

**Affiliations:** ^1^State Key Laboratory of Animal Nutrition, College of Animal Science and Technology, China Agricultural University, Beijing, China; ^2^College of Resources and Environmental Sciences, China Agricultural University, Beijing, China; ^3^Tianjin Zhongsheng Feed Co. Ltd., Tianjin, China

**Keywords:** antioxidant capability, fatty acids, *Forsythia suspensa* extract, meat quality, microbial community, finishing pigs

## Abstract

This study aimed to determine the effects of *Forsythia suspensa* extracts (FSE) on performance, antioxidant status, inflammatory cytokines, meat quality, meat fatty acid composition, and gut microbial community in finishing pigs. Sixty-four pigs [Duroc × (Landrace × Yorkshire)] with an average initial body weight of 88.68 kg were randomly allotted to two dietary treatments, with eight replicate pens per treatment (four pens were barrows and four pens were gilts), four pigs per pen. The dietary treatments included a corn–soybean meal basal diet (CON) and an FS diet (basal diet + 100 mg/kg FSE; FS). Compared with CON, pigs fed FSE showed enhanced (*P* < 0.05) saturated fatty acid (SFA)/polyunsaturated fatty acid (PUFA) ratio, reduced (*P* < 0.05) lightness, and *n*−6/*n*−3 PUFA ratio, as well as tended to increase C20:5n3 content in the longissimus dorsi muscle. Moreover, pigs fed FSE showed decreased (*P* < 0.05) serum cortisol and tumor nuclear factor-α contents, and increased (*P* < 0.05) serum high-density lipoprotein cholesterol, superoxide dismutase, and glutathione peroxidase contents compared with CON. These pigs also tended to have increased serum total protein and immunoglobulin G contents, and decreased serum low-density lipoprotein cholesterol and interleukin-1β contents compared with CON. In the colon, pigs fed FSE had a higher (*P* < 0.05) relative abundance of Bifidobacteriales at the order level, Lactobacillaceae and Bifidobacteriaceae at the family level, as well as *Lactobacillus* and *Bifidobacterium* at the genus level compared with CON. In conclusion, dietary *Forsythia suspensa* extract supplementation effectively improved antioxidant status and anti-inflammatory functions, as well as modulated meat fatty acid composition, and gut microbial community in finishing pigs.

## Introduction

In the pig industry, the growth performance, health status, and meat quality are crucial for growing–finishing pigs. Many studies showed Chinese herbal extracts as feed additives that have attracted wide attention in improving growth performance, digestive enzyme secretion, immune stimulation, antibacterial, and antiviral and antioxidant effects in growing-finishing pigs (1, 2). While several studies have also indicated that herb extract was beneficial for the intestinal microbial community, meat quality, and gut morphology in pigs (3, 4).

*Forsythia suspensa* is the one of Chinese natural herbs, which has strong antioxidant, antibacterial, antiviral, and anti-inflammatory properties (5). Previous studies in our lab have demonstrated that *F. suspensa* extract (FSE) improved the performance and nutrient digestibility of weaned piglets by improving antioxidant capacity, immune function, and intestinal morphology (6, 7). FSE could improve performance and antioxidant enzyme activities in broilers under heat or high-density-induced oxidative stress (8, 9). Furthermore, FSE as an antibiotic substitute could modulate the microbiota composition in the large intestine *via* increasing *Lactobacillus* and reducing the *Escherichia coli* (*E. coli*) community in broilers (10). Moreover, our previous studies showed that FSE could also increase the fatty acid deposition (especially *n*−3 polyunsaturated fatty acid content) in breast and thigh meat of broilers under the transportation and corticosterone-induced oxidative stress (11, 12).

However, to the best of our knowledge, there is little research focusing on the effect and possible functional mechanism of FSE on antioxidant status, immune function, gut health, and meat quality in finishing pigs. Based on the previous studies in our lab, we hypothesized that FSE might improve performance, health status, and meat quality *via* improving antioxidant status, anti-inflammatory function, meat fatty acid composition, and gut microbial community in finishing pigs. Therefore, this study was conducted to verify the hypothesis and provide valuable data for the application of FSE in modulating meat quality and the health status of finishing pigs.

## Materials and methods

The study was done at the Academician Workstation in Chengdejiuyun Agricultural and Livestock Co., Ltd. (FengNing Research Unit of China Agricultural University, Hebei, China). The animal procedures were agreed upon by the Institutional Animal Care and Use Committee of China Agricultural University (Beijing, China, AW21402202-1-2).

### Experimental products

The FSE used in this study was prepared and produced according to the study in our lab. Dried and ground forsythia fruits from Henan province were prepared and extracted with 80% ethanol, sonicated for 1 h, and then filtered. Ethanol was used to extract the residue twice and then rotary vaporization (Buchi, Rotavapor R-124, Flawil, Switzerland) was utilized to dry and combine the filtrates. And according to the data from the ^1^H-Nuclear Magnetic Resonance (NMR) and ^13^C-NRM in a previous study in our lab, the major active compounds of FSE were 33.0 mg/kg forsythoside A, 82.6 mg/kg forythialan A, 33.4 mg/kg phillygenin, and 163.4 mg/kg phillyrin.

### Animal, diets, and experimental design

A total of 64 pigs [Duroc × (Landrace × Yorkshire)] with an average initial body weight (BW) of 88.68 kg were randomly allotted to one of two dietary treatments, with eight replicate pens per treatment (four pens were barrows and four pens were gilts), four pigs per pen. The dietary treatments included a corn–soybean meal basal diet (CON) and an FS diet (basal diet + 100 mg/kg FSE; FS). The dose of FSE was determined according to a previous study (8, 9). The experiment ended until the pigs were sent for slaughter (about 110 kg). The experimental diets were formulated to meet or exceed the requirements suggested by the National Research Council 2012 (13). The feed ingredient and nutrient composition in the basal diet are presented in Table 1.

**Table 1:** Composition and nutrient levels of basal diets (%, as-fed basis).

**Items**	
Corn	83.47
Soybean meal	13.00
Soy oil	0.49
Dicalcium phosphate	0.83
Limestone	0.72
Salt	0.30
L-lysine	0.31
DL-methionine	0.02
L-threonine	0.08
L-tryptophan	0.03
Chromic oxide	0.25
Vitamin-mineral premix ^1^	0.50
**Calculated nutrient levels**	
Digestive energy, kcal/kg	3,402
Crude protein	12.47
Calcium	0.52
Digestible phosphorus	0.24
Standardized ileal digestible lysine	0.73
Standardized ileal digestible methionine	0.21
Standardized ileal digestible threonine	0.46
Standardized ileal digestible tryptophan	0.13

^1^ The premix provided the following compounds per kilogram of basal diet: vitamin A, 5200 IU; vitamin E, 17.2 IU; vitamin K_3_, 1.6 mg; vitamin D_3_, 2000 IU; vitamin B_2_, 3.6 mg; vitamin B_1_, 0.8 mg; vitamin B_12_, 10 μg; vitamin B_6_, 1.6 mg; pantothenic acid, 8.8 mg; nicotinic acid, 17.6 mg; biotin, 32 μg; folic acid, 0.38 mg; choline chloride, 0.24 g; Cu, 15 mg; Fe, 80 mg; Mn, 5 mg; Se, 0.3 mg; I, 0.2 mg.

Pigs were raised on the concrete floor and partially steel-slatted, and in pens (the size was 2.7 × 1.8 m) equipped with stainless steel feeders and nipple drinkers. These pigs had free access to water and feed, and the room temperature was kept at about 22°C. At the beginning and end of the experiment, pigs were weighed and the feed was recorded for the calculation of average daily feed intake (ADFI), average daily gain (ADG), and gain to feed ratio (ADG/ADFI, G: F).

### Sample collection and processing

On the end day of the experiment, feeders were taken from pens. Five pigs close to the average BW in each treatment from different pens were used for the collection of blood samples (approximately 10 mL) from the jugular vein using vacuum tubes (Becton Dickinson Vacutainer Systems, Franklin Lakes, NJ, USA) and subsequently centrifuged at 3,000 × *g* for 10 min at 4°C. Serum was withdrawn and stored at −80°C until further analysis.

Moreover, five pigs close to the average BW in each treatment were slaughtered after electrical stunning at the end day of the experiment. The live weight and the hot carcass weight (stripped of the head, hair, viscera, feet, and leaf fat) of pigs were recorded and the carcass yield was calculated as follows: hot carcass weight/live weight. The back fat thickness was measured *via* a vernier caliper at the midline of the 10 th rib, while the carcass oblique length was measured as the length from the 1 st rib to the aitch bone. The longissimus muscle area was calculated as follows: height × width × 0.7, while the section of intercostal space from the 10 th and 11 th ribs was measured as the longissimus dorsi muscle height and width (14). Moreover, the left longissimus dorsi muscle (between the 10 th and 11 th ribs) was collected and divided into two parts, one part was kept at 4°C for the analysis of meat color, drip loss, and pH values, and another part was stored at −80°C for the analysis of fatty acid composition. The colon digesta samples (about 100 g) were frozen by liquid nitrogen immediately and stored at −80°C for the analysis of the microbial community.

### Determination of meat quality

A glass penetration pH electrode (pH-star, Matthaus, Germany) was used for the measurement of the pH values at 24 h of the longissimus dorsi muscle, while the Chromameter (CR-410, Konica Minolta, Tokyo, Japan) was used for the measurement of meat color, including lightness (L^*^), redness (a^*^), and yellowness (b^*^) value. The drip loss at 24 h of the longissimus dorsi muscle was measured *via* the plastic bag method as described by Long et al. (15).

### Measurement of serum biochemical parameters

Following the instructions of the manufacturer of the corresponding reagent kit (Zhongsheng Biochemical Co. Ltd., Beijing, China), the contents of glucose, total protein (TP), urea, total cholesterol (TC), low-density lipoprotein cholesterol (LDLC), high-density lipoprotein cholesterol (HDLC), and cortisol in serum of pigs were analyzed using colorimetric methods by an automatic biochemical analyzer (RA-1000, Bayer Corp., Tarrytown, NY, USA). While the contents of immunoglobulins (IgA, IgG, and IgM) in the serum of pigs were analyzed with immuno-turbidimetry using immunoglobulin-specific kits (Sanwei Biological Engineering Co., Ltd., Shandong, China). The commercially available kits (Nanjing Jiancheng Biological Product Co., Ltd., Nanjing, China) were used to measure the contents of total-antioxidant capability (T-AOC), malondialdehyde (MDA), superoxide dismutase (SOD), glutathione peroxidase (GSH-Px), tumor nuclear factor-α (TNF-α), interleukin-1β (IL-1β) in serum.

### Fatty acid profile of the longissimus dorsi muscle

The longissimus dorsi muscle samples were defrosted lyophilized for 72 h using a freeze dryer. Then, the total lipids of the muscle samples were extracted by a solvent mixture of chloroform and methanol (2:1, vol/vol). The fatty acid profile in muscle was measured using gas chromatography (GC, 6890 series, Agilent Technologies, Wilmington, DE, USA) equipped with a capillary column (60 m × 250 m × 250 nm, DB-23, Agilent) following the procedure used by Long et al. (16, 17). The fatty acid composition was calculated in terms of mg/g tissue of muscle (dry matter base). The saturated fatty acid (SFA), monounsaturated fatty acid (MUFA), polyunsaturated fatty acid (PUFA), *n*−6 PUFA, *n*−3 PUFA, and *n*−6:*n*−3 PUFA ratio. *n*−6 PUFA, *n*−3 PUFA, the *n*−6/n-3 PUFA ratio, and PUFA/SFA ratio (P/S) in the current study were calculated as follows: *N*−6 PUFA: C18:2 *n*−6+C18:3 *n*−6+ C20:2 + C20:3*n*6 + C20:4 *n*−6; *N*−3 PUFA: C18:3 *n*−3 + C20:5 *n*−3+C22:6 *n*−3; SFA: C 10:0 + C12:0 + C14:0+C16:0+C17:0+C18:0+C20:0 + C21:0 + C22:0 + C24:0; MUFA: C18:1*n*9c + C20:1 + C22:1*n*9 + C24:1; PUFA: C18:2 *n*−6+C18:3 *n*−6+ C18:3 *n*−3 + C20:2 + C20:3*n*6 + C20:4 *n*−6+ C20:3*n*3 + C20:5 *n*−3+C22:6 *n*−3.

### Analysis of bacterial microbiota by 16S RNA sequences

The DNA was extracted from colon digesta samples (*n* = 4) of pigs using the bacterial DNA kit (Omega Bio-Tek Inc., Norcross, GA, USA). The DNA concentration and purity were preliminarily evaluated by using the Shimadzu spectrophotometer, using the barcoded primers 338F (5′-ACTCCTACGGGAGGCAGCAG-3′) and 806R (5′-GGACTACHVGGGTWTCTAAT-3′) to amplify the V3-V4 hypervariable regions of the bacterial 16S rRNA genes. The amplified library was sequenced for paired-end reads of 300 bp on the Illumina Hiseq PE250 platform (Illumina, San Diego, USA). Paired-end reads were assembled into longer tags and quality-filtered to remove tags with an average quality score of < 20, a length of < 220 bp, and tags containing >3 ambiguous bases by PANDAseq. After discarding the singletons, the high-quality tags were clustered into operational classification units (OTUs) using Quantitative Insights Into Microbial Ecology (QIIME) pipeline software (version 1.8.0) (a similarity threshold of 0.97). The RDP database (https://rdp.cme.msu.edu/) was used for the further analysis of the OTUs (18), while the linear discriminant analysis (LDA) was used to perform the effect size analysis (LEfSe). The relative abundance of bacteria was expressed as a percentage (19), while the QIIME was used for the analysis of the alpha and beta diversities (20). The data presented in the study are deposited in the NCBI repository, accession number PRJNA848278.

### Statistical analysis

Using GLM models of SAS 9.2 (2008) (21) and *Students' t*-*tests* were used to analyze the experimental data, and the results were shown as mean values ± SEM. For performance, each pen was used as an experimental unit, while for other parameters, each pig was used as an experimental unit. The standardized OTUs reads were used to analyze bacterial diversity with the guidance of the R software. The abundance of bacteria at the phylum and genus levels were shown as bar plots. The population of the bacterial community in digesta samples at the phyla, families, and genera levels was analyzed by the method of the Kruskal-Wallis rank sum test. The abundances of differential bacteria were classified using the procedure of the LDA effect size (LEfSe) algorithm if the logarithmic LDA values of bacteria exceeded 2.0. A significant difference was defined at *P* ≤ 0.05, while a tendency for the significance was defined at 0.05 < *P* ≤ 0.10.

## Results

### Growth performance

As shown in Table 2, pigs fed FSE showed increased ADG at 9%, ADFI at 1%, and G: F at 7% compared with CON. However, there is no significant difference among treatments.

**Table 2:** Effect of *Forsythia suspensa* extract on the performance of finishing pigs.

**Items**	**CON^1^**	**FSE^1^**	**SEM**	***P*-Value**
Initial body weight, kg	88.58	88.77	0.15	0.47
Final body weight, kg	114	116	1.44	0.36
Average daily gain, g	847	920	45.27	0.37
Average daily feed intake, g	2,923	2,998	209.21	0.82
Gain to feed ratio	0.29	0.31	0.02	0.51

SEM means standard error of the mean. ^1^ CON, control; FSE, *Forsythia suspensa* extract.

### Meat quality

As shown in Table 3, pigs fed FSE showed reduced (*P* < 0.05) lightness in the longissimus dorsi muscle compared with CON. However, there was no significant difference in hot carcass weight, carcass yield, carcass oblique length, back fat thickness, longissimus muscle area, drip loss, and pH value between the two treatments.

**Table 3:** Effect of *Forsythia suspensa* extract on the meat quality of finishing pigs.

**Items**	**CON^1^**	**FSE^1^**	**SEM**	***P*-Value**
Carcass yield (kg/100 kg live weight)	73.16	73.18	0.36	0.60
Carcass oblique length (cm)	132.80	127.20	2.10	0.13
Back fat thickness (cm)	1.56	1.66	0.22	0.77
Longissimus muscle area (cm^2^)	68.36	71.81	5.89	0.70
Lightness (L*)	60.55^a^	54.48^b^	1.45	0.04
Redness (a*)	17.24	17.40	0.37	0.77
Yellowness (b*)	8.97	5.50	1.54	0.19
Drip loss, %	7.00	7.60	0.35	0.32
pH 24 h	5.42	5.42	0.02	0.95

SEM means standard error of the mean. *n* = 5. ^a − b^ Different superscripts within a row indicate a significant difference (*P* < 0.05). ^1^ CON, control; FSE, *Forsythia suspensa* extract.

### Meat fatty acid composition

As shown in Table 4, pig fed FSE showed increased (*P* < 0.05) C14:1, C22:1*n*9 contents and SFA/PUFA ratio, decreased (*P* < 0.05) *n*−6/*n*−3 PUFA ratio, as well as tended to have increased C10:0 and C20:5*n*3 contents in the longissimus muscle compared with CON.

**Table 4:** Effect of *Forsythia suspensa* extract on the fatty acid composition in the longissimus dorsi muscle of finishing pigs (mg/g tissue of muscle, dry matter base).

**Items**	**CON^1^**	**FSE^1^**	**SEM**	***P*-Value**
C10:0	0.10	0.12	0.00	0.08
C12:0	0.04	0.06	0.01	0.15
C14:0	0.54	0.95	0.16	0.14
C14:1	0.01^b^	0.02^a^	0.00	0.02
C15:0	0.01	0.02	0.00	0.48
C16:0	10.08	17.26	3.11	0.18
C16:1	1.19	1.73	0.23	0.17
C17:0	0.06	0.09	0.02	0.40
C18:0	5.89	10.19	2.21	0.24
C18:1n9c	16.29	27.30	4.53	0.16
C18:2n6c	3.89	5.46	0.93	0.30
C18:3n3	0.11	0.19	0.05	0.28
C20:0	0.09	0.16	0.03	0.18
C20:1	0.30	0.51	0.10	0.23
C21:0	0.16	0.25	0.05	0.29
C20:2	0.03	0.03	0.00	0.62
C20:3n6	0.12	0.11	0.01	0.53
C20:4n6	0.77	0.64	0.06	0.18
C20:3n3	0.02	0.04	0.00	0.24
C22:0	0.02	0.02	0.00	0.37
C20:5n3	0.03	0.04	0.00	0.09
C22:1n9	0.02^b^	0.03^a^	0.00	0.03
C24:0	0.02	0.02	0.00	1.00
C24:1	0.02	0.02	0.00	0.18
C22:6n3	0.06	0.06	0.00	0.82
Saturated fatty acid (SFA)	17.03	29.13	5.59	0.20
Monounsaturated fatty acid (MUFA)	17.84	29.61	4.85	0.16
Polyunsaturated fatty acid (PUFA)	5.01	6.54	1.00	0.34
n-6 PUFA	4.78	6.21	0.95	0.35
n-3 PUFA	0.23	0.33	0.05	0.25
SFA/PUFA	3.27^b^	4.28^a^	0.25	0.05
n-6/n-3 PUFA	20.59^a^	19.01^b^	0.35	0.03

SEM means standard error of the mean. *n* = 5. ^a − b^Different superscripts within a row indicate a significant difference (*P* < 0.05). ^1^CON, control; FSE, *Forsythia suspensa* extract.

*N*−6 PUFA: C18:2*n*−6+C18:3 *n*−6+ C20:2 + C20:3*n*6 + C20:4 *n*−6.

*N*−3 PUFA: C18:3 *n*−3+C20:5 *n*−3+C22:6 *n*−3.

SFA: C 10:0 + C12:0 + C14:0+C16:0+C17:0+C18:0+C20:0 + C21:0 + C22:0 + C24:0.

MUFA: C18:1*n*9c + C20:1 + C22:1*n*9 + C24:1.

PUFA: C18:2n-6+C18:3n-6+ C18:3n-3 + C20:2 + C20:3n6 + C20:4n-6+ C20:3n3 + C20:5n-3+C22:6n-3.

### Biochemical indicators, antioxidant status, and immune function

As shown in Table 5, pigs fed FSE had decreased (*P* < 0.05) cortisol content, and increased (*P* < 0.05) HDLC content, as well as tended to have increased (*P* = 0.06) TP content and decreased (*P* = 0.06) LDLC content in serum compared with CON.

**Table 5:** Effect of *Forsythia suspensa* extract on serum biochemical indicators of finishing pigs.

**Items**	**CON^1^**	**FSE^1^**	**SEM**	***P*-Value**
Glucose (mmol/L)	4.05	4.46	0.21	0.24
Total protein (g/L)	52.20	54.75	1.80	0.06
Urea (mmol/L)	1.39	1.34	0.12	0.79
Total cholesterol (mmol/L)	1.85	2.02	0.23	0.28
Triglyceride (mmol/L)	0.35	0.38	0.06	0.89
Low-density lipoprotein cholesterol (mmol/L)	0.88	0.71	0.05	0.06
High-density lipoprotein cholesterol (mmol/L)	0.93^b^	1.01^a^	0.05	0.03
Cortisol (nmol/L)	86.22^a^	71.20^b^	4.20	0.04

Note: SEM means standard error of the mean. *n* = 5.

^a − b^ Different superscripts within a row indicate a significant difference (*P* < 0.05).

^1^ CON: control; FSE: *Forsythia suspensa* extract.

As shown in Table 6, pigs fed FSE showed increased (*P* ≤ 0.05) SOD and GSH-Px content in serum compared with CON.

**Table 6:** Effect of *Forsythia suspensa* extract on the antioxidant status of finishing pigs.

**Items**	**CON^1^**	**FSE^1^**	**SEM**	***P*-Value**
**Serum**				
Total antioxidant capacity (mmol/L)	0.24	0.24	0.02	1.00
Superoxide dismutase (U/mL)	134.38	159.99	5.00	0.05
Glutathione peroxidase (U/mL)	445.53^b^	467.63^a^	5.21	0.05
Malondialdehyde (nmol/mL)	4.26	4.15	0.27	0.78

SEM means standard error of the mean. *n* = 5.

^a − b^ Different superscripts within a row indicate a significant difference (*P* < 0.05).

^1^ CON: control; FSE: *Forsythia suspensa* extract.

As shown in Table 7, pigs fed FSE showed decreased (*P* < 0.05) TNF-α content, and tended to have decreased (*P* = 0.07) IL-1β content and increased (*P* = 0.10) IgG content in serum compared with CON.

**Table 7:** Effect of *Forsythia suspensa* extract on serum immunoglobulin and interleukin cytokines in finishing pigs.

**Items**	**CON^1^**	**FSE^1^**	**SEM**	***P*-Value**
IgG (g/L)	5.29	6.37	0.38	0.10
IgM (g/L)	0.74	0.78	0.03	0.45
IgA (g/L)	0.82	0.87	0.08	0.65
TNF-α (pg/mL)	176.22^a^	140.02^b^	7.89	0.03
IL-1β (pg/ mL)	16.59	12.93	1.06	0.07

SEM means standard error of the mean. *n* = 5. ^a − b^ Different superscripts within a row indicate a significant difference (*P* < 0.05). ^1^ CON: control; FSE: *Forsythia suspensa* extract.

### Intestinal microbial community

**Figure 1:**
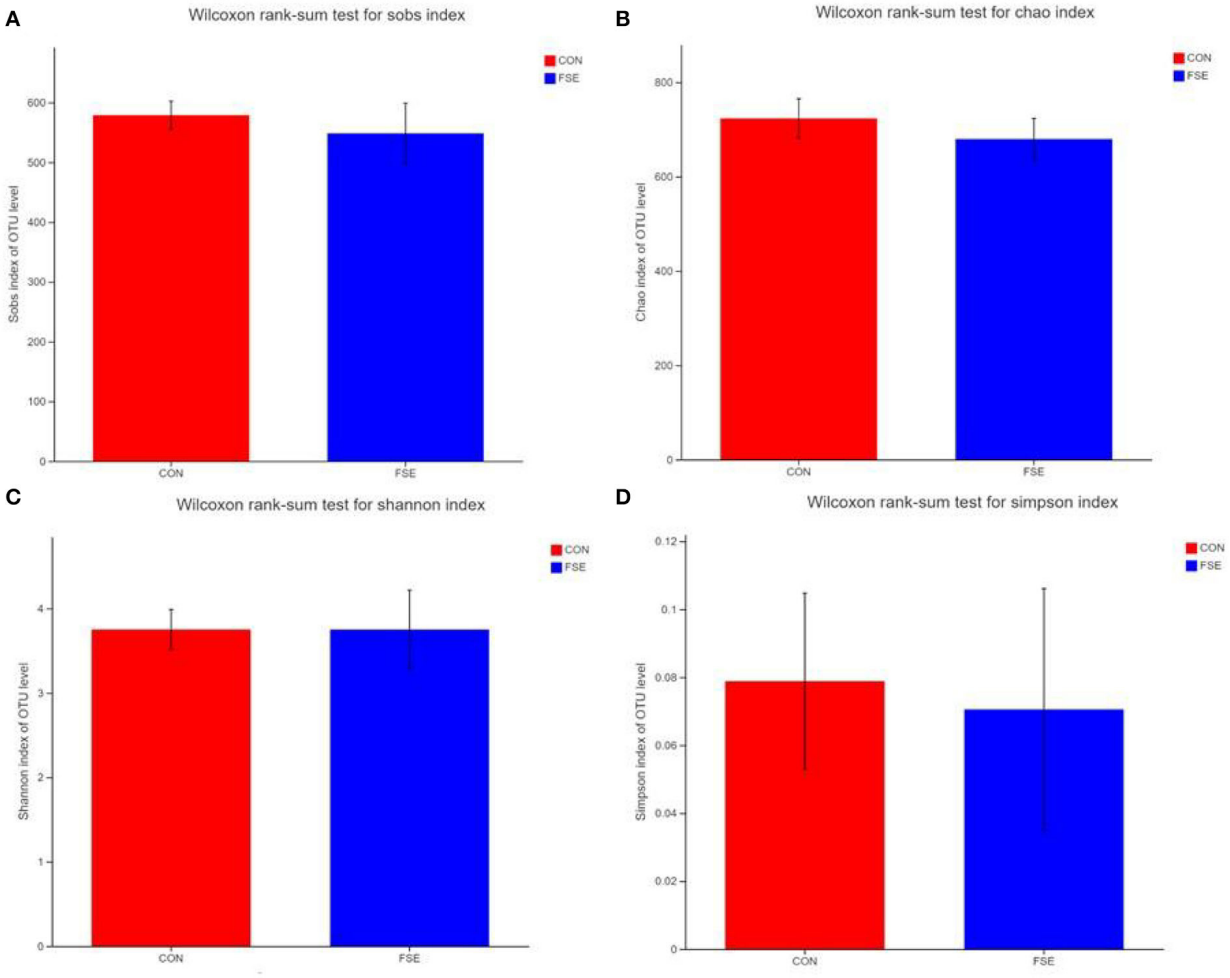
Effects of *Forsythia suspensa* extract on α-diversity in the colon digesta of finishing pigs **(A)** Wilcoxon rank sum test for sob index; **(B)** Wilcoxon rank sum test for Chao index; **(C)** Wilcoxon rank sum test for the Shannon index; **(D)** Wilcoxon rank sum test for the Simpson index; CON: control; FSE: *Forsythia suspensa* extract. *n* = 4.

**Figure 2:**
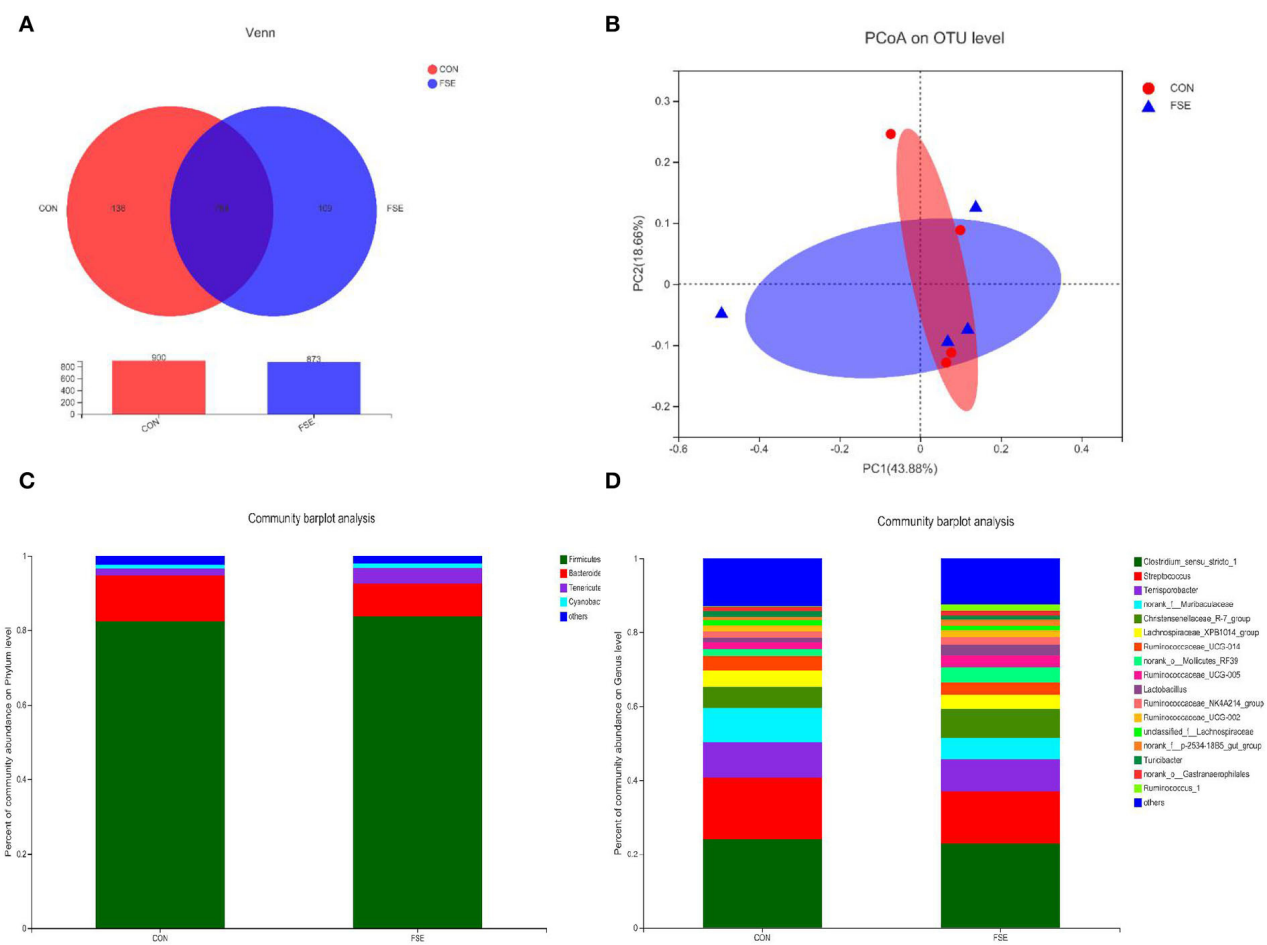
The microbial community composition of colon digesta in finishing pigs supplemented with FSE **(A)** Venn diagrams of the bacterial operational taxonomic units (OUT) community among treatments; **(B)** PCoA on OUT level of microbial community among treatments; **(C)** Distribution of community barplot of microbial community at phylum level with the relative abundance higher than 0.01% among treatments; **(D)** Distribution of community barplot of microbial community at genus level with the relative abundance higher than 0.01% among treatments. CON: control; FSE: *Forsythia suspensa* extract. *n* = 4.

According to Figure 1, there were no differences (*P* > 0.05) in α-diversity (including Sobs, Shannon, Simpson, and Chao indexes) in the colon digesta of finishing pigs. As shown in Figure 2, the bacterial OTUs community of CON and FSE were 900 and 873, respectively (Figure 2A). There were no differences in the β-diversity of the microbial community in the colon digesta of finishing pigs (Figure 2B). The relative abundance of main microbiota in colon digesta of pigs fed CON and FSE were Firmicutes, Bacteroidetes, Tenericutes, and Cyanobacteria (Figure 2C). And the relative abundance of main microbiota at genus level in colon digesta between CON and FSE were *Clostridium_sensu_sricto_1, Streptococcus, Terrisporobacter, norank_f_Muribaculaceae, Christensenellaceae_R-7_group, Lactobacillus*, and *Ruminococcaceae_UCG-005* (Figure 2D).

**Figure 3:**
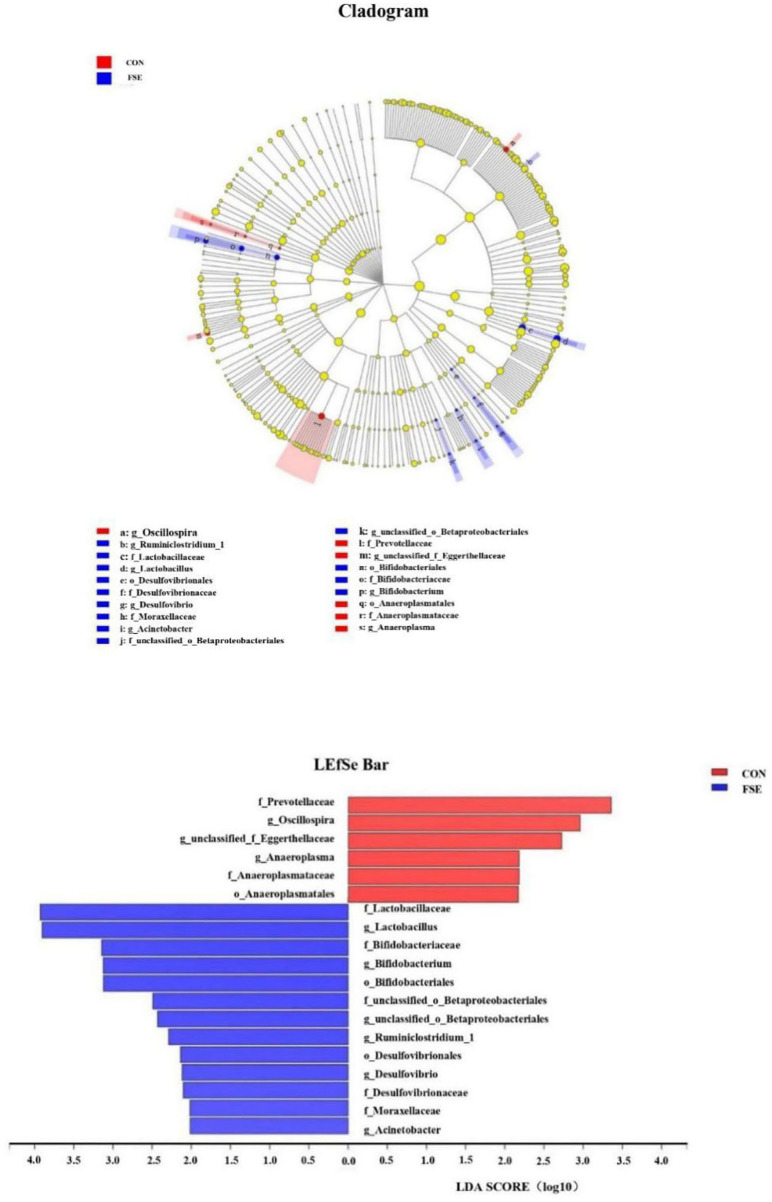
Key phylotypes of microbiota communities in colon digesta of finishing pigs fed basal diets (CON) or basal diet with *Forsythia suspensa* extract (FSE) were identified using linear discriminant analysis effect size (LEfSe) analyses. Taxonomic differences between FSE and CON are represented by the color of the most abundant class (red for CON, blue for FSE). The diameter of each circle represents the relative abundance of the taxon. The central point represents the root of the tree (Bacteria), and each ring represents the next lower taxonomic level (phylum to genus: p, phylum; c, class; o, order; f, family; g, genus). LEfSe analysis used the Kruskal-Wallis rank sum test to detect significantly different abundances and performs LDA scores to estimate the effect size (Threshold: ≥2). *n* = 4.

According to Figure 3, pigs fed FSE had a higher (*P* < 0.05) relative abundance of Bifidobacteriales and Desulfovibrionales at the order level, Moraxellaceae, Lactobacillaceae, Bifidobacteriaceae, Desulfovibrionaceae, and unclassified_o__Betaproteobacteriales at the family level, as well as *Lactobacillus, Bifidobacterium, Acinetobacter, Ruminiclostridium_1, Desulfovibrio*, and *unclassified_o__Betaproteobacteriales* at the genus level. Moreover, pigs fed FSE had a lower (*P* < 0.05) relative abundance of Anaeroplasmatales at the order level, Eggerthellaceae, Prevotellaceae, and Anaeroplasmataceae at the family level, as well as *Oscillospira* and *Anaeroplasma* at the genus level.

## Discussion

At the finishing stage of pigs, the final body weight, meat quality, and health status are very important for commercial issues. In the current study, we found that dietary FSE supplementation in finishing pigs numerically increased the ADG and G:F, but the rate was not a significant difference, indicating FSE might have the potential to improve performance. Plant extracts were often used as feed additives in pigs, Janz et al. (22) found that 0.05% fragrant plant extracts from rosemary, garlic, ginger, and mint could significantly increase ADG and G: F in fattening pigs. Zhou et al. (23) also reported that the dietary Chinese *Coptis chinensis* herb extract supplementation improved the performance and nutrient digestibility in growing–finishing pigs. However, according to the study by Hanczakowska et al. (24), dietary herb extracts supplementation showed no significant difference in the performance of finishing pigs, which was in agreement with the result of the current study. These contradictory results regarding the performance responses in different studies might be due to the differences in herb species and concentrations, and the physiological phases of pigs. In previous studies from our lab, we found that dietary supplementation with 100 mg/kg or 200 mg/kg FSE could significantly improve performance (ADG and G:F) in weaned piglets (6, 7) *via* promoting early intestinal development and regulating the structure of intestinal flora, which indicated that the growth promoting the ability of FSE was usually showed in young animals. However, for the finishing pigs, the intestinal development has been completed, which might be the reason that FSE did not promote growth performance in finishing pigs. Another reason for the current finding might be that the experiment phase was not enough for FSE to play a cumulative effect on performance in pigs (7).

Lightness is one of the indicators to measure meat quality. Zhou et al. (23) reported that herb extract (*C. chinensis*) could improve meat quality in growing–finishing pigs. The current study found that FSE significantly reduced lightness, along with the increased antioxidant capacity in the longissimus dorsi muscle, indicating that meat quality was potentially improved by FSE. This finding was in agreement with Lan et al. (25), who reported that dietary herb supplementation (*Astragalus membranaceus, Codonopsis pilosula*, and allicin mixture) increased redness values and decreased lightness value in meat. However, dietary carob supplementation did not affect meat color stability in pigs (26). These inconsistent findings about meat color might also be due to the different types of herbs used in different studies. In this study, we did not find any significant difference in carcass yield, carcass oblique length, back fat thickness, longissimus muscle area, and drop loss between the two treatments in finishing pigs, which was in agreement with the study of Janz et al. (22), who reported that plant extracts had no effect on carcass weight, slaughter rate, and back fat thickness of pigs. The finding indicated that the effect of plant extracts on carcass traits might be a slow process. Therefore, one of the possible reasons for this finding might be that the experiment time was not enough for FSE to play a cumulative effect on the carcass trait in pigs.

Stress response induces physiological, behavioral, immunological, and biochemical changes that directly affect the health status of pigs. The current study showed the addition of FSE significantly reduced serum cortisol in finishing pigs, which was in agreement with the study of Casal et al. (27), who reported that an herbal compound could reduce stress in growing pigs. Wang et al. (8) demonstrated that dietary FSE supplementation could effectively alleviate oxidative damage to broilers under heat stress, indicating that FSE could also have a similar function in finishing pigs. The current study also found FSE increased HDLC content, as well as tended to increase TP content and decrease LDLC content in serum compared with CON. Serum concentrations of TP reflected the functions of protein synthesis in pigs. The increased rate of triglyceride synthesis in the liver could exceed the capacity to produce very-low-density lipoproteins. An increased HDLC content and a tendency of decreased LDLC in the serum of pigs reflected that fatty acid utilization and metabolism were improved by FSE, which was beneficial for the health status of pigs and the reduction of the risk of diseases (15, 28).

Immunoglobulins, which mainly exist in serum, play an important role in reacting with antigens and increasing antibacterial and antiviral capabilities in pigs. IgG is the main antibody component in blood and has important immunological effects. The current study also found pigs fed FSE tended to increase serum IgG levels compared with CON, which was partly in agreement with the study of Wang et al. (29), who reported that dietary herbs and coral mineral complex supplementation could increase serum IgG contents in growing pigs. This finding might be due to the herb extract enhanced blood erythrocytes counts, immune function, and health status of the gastrointestinal environment in growing–finishing pigs (4, 24, 30). Moreover, this finding might be also due to the phillyrin, forythialan A, phillygenin, and forsythoside A in FSE playing an important role in improving immune responses (8, 9). The TNF-α and IL-1β were pro-inflammatory cytokines. The current study showed FSE decreased or tended to decrease serum TNF-α and IL-1β contents in finishing pigs, indicating that the pro-inflammatory response of pigs was decreased and the anti-inflammatory function was improved by FSE. One of the possible reasons might be that the phillyrin in FSE could inhibit the LPS-induced inflammatory damage and increase immune response, which benefited the health status and performance (31). Moreover, the current finding might also be due to the beneficial effect of forsythoside A in FSE, since plenty of *in vivo* and *in vitro* studies have demonstrated that forsythoside A played important role in reducing pro-inflammatory cytokines and improving anti-inflammatory function *via* inhibiting the activation of nuclear factor kappa B (NF-κB) (5, 32). Furthermore, this finding might also be due to the improved antioxidant status (including antioxidant enzyme activities) by FSE in the current study.

Under normal circumstances, the metabolism of free radicals in pigs is in a dynamic balance, while stress could cause excessive generation of oxygen free radicals, which may lead to lipid oxidation, and protein and DNA degradation in pigs (33). Pan et al. (11) pointed out that transportation-induced oxidative stress might reduce the antioxidant capacity of animals and animal meat products. Previous studies in our lab also showed FSE reduced oxidative stress and enhanced performance under heat stress and corticosterone stimulation in broilers (8, 34). For finishing pigs, the transportation-induced oxidative stress might influence lipid oxidation, meat fatty acid profiles, and meat stability. The enzymatic antioxidant system includes SOD, GSH-Px, and CAT. The SOD can scavenge superoxide anion free radicals and eliminate their damage to cell membranes. The GSH-Px and CAT can convert hydrogen peroxide (H_2_O_2_) into H_2_O. The MDA is the main product of lipid peroxide degradation. In this study, pigs fed FSE showed increased GSH-Px content in serum, which was partly in agreement with Liu et al. (35), who found that the polyphenolic compounds in *S. baicalensis* and *Lonicera japonica* extract could decrease TBARS and lipid oxidation in meat. Similarly, previous studies also showed dietary 500 mg/kg herbal extracts (sage, nettle, lemon balm, and coneflower) supplementation had a beneficial effect on reducing lipid peroxidation and the level of MDA in pork (25, 36). However, dietary carob supplementation increased meat susceptibility to lipid oxidation across storage (26). The differences in these findings might be due to the differences in the amount of active ingredients of plant extracts, and the age or types of animals used in different studies. The reason for the current finding might be due to the beneficial effect of the polyphenolic compounds (forsythoside A, forythialan A, phillygenin, and phillyrin) in FSE on exhibiting antioxidant properties and scavenging free radicals and eliminating oxidative damage in pigs (33, 37). Furthermore, the forsythialan A in FSE had aromatic hydroxyl, while phillygenin in FSE had hydroxy substituents on the phenyl moieties, which could help alleviate oxidative stress and improve antioxidant status in pigs (38, 39). In addition, the ursolic acid, phillygenin, and rutin in FSE could also help decrease oxidative-stress-related lipid peroxidation and eliminate α,α-Diphenyl-β-picrylhydrazyl radical (DPPH) *in vitro* (40), which might be beneficial for improving antioxidant status in pigs.

Meat is a good source of fatty acids in human diets. There has been a growing interest to manipulate the fatty acid composition of meat through diets. Ahmed et al. (28) suggested that a herb combination (pomegranate, Ginkgp biloba, licorice) in natural or fermented form could modify the fatty acid composition in the longissimus thoracis muscle. The current study showed FSE could decrease the *n*−6/*n*−3 PUFA ratio and tend to increase C20:5*n*3 (Eicosapentaenoic acid, belonging to *n*−3 PUFA), indicating that the FSE has the potential on improving the fatty acid composition of the longissimus dorsi muscle in finishing pigs and reducing the risk of diseases (15, 28). This finding indicates pork from FSE-fed pigs is beneficial for human consumption and health status. Zhou et al. (23) reported that dietary *C. chinensis* herb extract supplementation increased the content of total unsaturated fatty acids in the longissimus dorsi muscle of finishing pigs, and Hanczakowska et al. (24) reported that herbal extracts (*Salvia officinalis, Urtica dioica, Melissa officinalis, Echinacea purpurea*) had a beneficial effect on increasing PUFA concentration in the longissimus dorsi muscle. However, the current study did not find any significant differences in PUFA concentration in the longissimus dorsi muscle, which might be due to the differences in herb types used in different studies. Feeding carb-containing diets reduced the concentration of saturated fatty acids in the muscle, increased the concentration of monounsaturated fatty acids in meat, and reduced the *n*−6/*n*−3 PUFA ratio (26). The reason for the increased C20:5*n*3 and decreased the *n*−6/*n*−3 PUFA ratio might be due to the phenolic compounds in FSE positively could modify the fatty acid composition by preventing the oxidation of unsaturated fatty acids (28, 41, 42). However, further study is still warranted to investigate the exact mechanism of the effects of FSE supplementation on fatty acid composition in the meat of pigs.

A previous study showed that dietary supplementation with soy protein concentrate could enhance short-chain fatty acid-producing microbes in broilers (Faecalibacterium and Marvinbryantia) and short-chain fatty acids, especially butyric acid in broilers (43). While dietary supplementation with dimethylglycine as a feed additive could improve host health by reducing the occurrence of intestinal inflammation and modulating cecal microbial communities (44). Recently, the herb has been used as a feed additive for improving the gut microbial community in livestock. Traditional herbal medicine could regulate the gut microbiota balance and the fermentation products of the gut microbes (45). Herb extracts, especially clove, could increase the stability of pork and reduce the harmful bacteria community (46). While FSE could selectively repress the growth of harmful bacteria and improved the growth of beneficial bacteria (47), which could maintain the intestinal microbiota balance of pigs. In the current study, pigs fed FSE had higher antioxidant status in pork, and a relative abundance of beneficial bacteria, including *Lactobacillus* and *Bifidobacterium* at the genus level in the colon. *Lactobacillus* and *Bifidobacterium* are crucial to the health status for providing metabolic, immunologic, and protective functions (3). The *Lactobacillus* (belongs to Firmicutes) is beneficial for modulating intestinal health *via* improving intestinal barrier function and immunity (31), which could help explain the improved immunity (increased IgG level) and anti-inflammatory functions (decreased TNF-α and IL-1β levels) in the current study. Moreover, *Lactobacillus* or *Bifidobacteria* can also produce some metabolites, including organic acids, such as lactic acid, formic acid, acetic acid, propionic acid, and butyric acid, which could inhibit a variety of pathogenic bacteria growth and reproduction without affecting the surrounding probiotics, and ultimately establish a micro-ecological environment with probiotics as the dominant position in the intestine (48). In addition, *Lactobacillus* can also metabolize enzyme components into SOD and CAT, which have significant antioxidant properties (49). In our previous study, we also found maternal FSE supplementation could increase *Lactobacillus* levels in the large intestine of weaned pigs (50), which revealed that FSE might modulate gut health by modulating the *Lactobacillus* community. The present finding might be due to the *Forsythoside* A, lignans, and glycosides in FSE could promote the proliferation of *Lactobacillus* and *Bifidobacteria* (51). A healthy and stable microbial environment could prevent the occurrence of intestinal diseases and result in improved performance (52). Further study is still warranted to investigate the mechanism of FSE on microbiota composition in the large intestine of finishing pigs.

## Conclusion

The current study demonstrated that dietary *F. suspensa* extracts' supplementation effectively improved antioxidant status and anti-inflammatory functions. Moreover, *Forsythia suspensa* extracts also modulated meat fatty acid composition, and gut microbial community by increasing the relative abundance of *Lactobacillus* and *Bifidobacteria* at the genus level in finishing pigs.

## Data availability statement

The original contributions presented in the study are included in the article/supplementary material, further inquiries can be directed to the corresponding author/s.

## Ethics statement

The animal study was reviewed and approved by Institutional Animal Care and Use Committee of China Agricultural University (Beijing, China, AW21402202-1-2).

## Author contributions

SLi, SLo, and XP conceived and designed the experiments. QW, JW, SLi, and JM performed the animal experiments and wrote the manuscript. HW, LL, and JW analyzed the data. XP supervised and provided continuous guidance for the experiments. All authors have discussed the results and reviewed the manuscript.

## Funding

This work was supported by the Beijing Municipal Natural Science Foundation (6202019) and the National Key Research and Development Program of China (2021YFD1300201).

## Conflict of interest

Author LL was employed by Tianjin Zhongsheng Feed Co. Ltd.

The remaining authors declare that the research was conducted in the absence of any commercial or financial relationships that could be construed as a potential conflict of interest.

## Publisher's note

All claims expressed in this article are solely those of the authors and do not necessarily represent those of their affiliated organizations, or those of the publisher, the editors and the reviewers. Any product that may be evaluated in this article, or claim that may be made by its manufacturer, is not guaranteed or endorsed by the publisher.
